# Antitumor Effect of 5-Fluorouracil-Loaded Liposomes Containing n-3 Polyunsaturated Fatty Acids in Two Different Colorectal Cancer Cell Lines

**DOI:** 10.1208/s12249-020-01897-5

**Published:** 2021-01-06

**Authors:** Yves Marc Dupertuis, Nathalie Boulens, Emmanuelle Angibaud, Anna-Sophia Briod, Alexandre Viglione, Eric Allémann, Florence Delie, Claude Pichard

**Affiliations:** 1 Clinical Nutrition, Geneva University Hospitals, Rue Gabrielle-Perret-Gentil 4, 1211 Geneva 14, Switzerland; 2 School of Pharmaceutical Sciences, Institute of Pharmaceutical Sciences of Western Switzerland, University of Geneva, Rue Michel-Servet 1, 1211 Geneva, Switzerland

**Keywords:** colorectal cancer, chemotherapy, 5-fluorouracil, polyunsaturated fatty acids, liposomes

## Abstract

It has been shown that long-chain n-3 polyunsaturated fatty acids (n-3 PUFAs) could act synergistically with 5-fluorouracil (5-FU) to kill cancer cells. To facilitate their simultaneous transport in the bloodstream, we synthesized, for the first time, liposomes (LIPUFU) containing 5-FU in the aqueous core and docosahexaenoic acid (DHA)/eicosapentaenoic acid (EPA) at a ratio of 1:2 in the lipid bilayer. LIPUFU werestable with uniform size of 154 ± 4 nm, PDI of 0.19 ± 0.03 and zeta potential of -41 ± 2 mV. They contained 557 ± 210 μmol/l DHA, 1467 ± 362 μmol/l EPA, and 9.8 ± 1.1 μmol/l 5-FU. Control liposomes without (LIP) or with only 5-FU (LIFU) or n-3 PUFAs (LIPU) were produced in a similar way. The effects of these different liposomal formulations on the cell cycle, growth, and apoptosis were evaluated in two human colorectal cancer (CRC) cell lines differing in sensitivity to 5-FU, using fluorescence-activated cell sorting analyses. LIPUFU were more cytotoxic than LIP, LIFU, and LIPU in both LS174T (p53^+/+^, bax^−/−^) and HT-29 (p53^−/0^, bax^+/+^) cell lines. Similar to LIFU, LIPUFU increased the percentage of cells in S phase, apoptosis, and/or necrosis. The cytotoxic potential of LIPUFU was confirmed *in vivo* by tumor growth inhibition in the chicken chorioallantoic membrane model. These results suggest that LIPUFU could be considered to facilitate the simultaneous transport of 5-FU and n-3 PUFAs to the tumor site, in particular in case of CRC liver metastases.

## INTRODUCTION

Colorectal cancer (CRC) is the third most commonly diagnosed malignancy and the second leading cause of cancer death (1, 2). Moreover, CRC incidence and mortality are constantly increasing worldwide because of the aging population and the adoption of harmful Western diet and sedentary lifestyle (1). Therefore, CRC prevention, screening, and treatment are among the main public health concerns. If CRC is diagnosed in the early stages by colonoscopy or sigmoidoscopy, complete cure can be obtained by surgical resection of the tumor with sufficient margins (3). However, when the disease has reached stage III/IV and spread to the lymph nodes or distant organs, adjuvant chemotherapy is required to prevent local recurrence and metastatic invasion (2). The reference drug in CRC treatment is 5-fluorouracil (5-FU), an antimetabolite that causes cell cycle arrest in S phase after conversion into fluoronucleotides and misincorporation into RNA and DNA (4). Usually, 5-FU is co-administrated with folinic acid, oxaliplatin, and/or irinotecan as FOLFOX, FOLFIRI, or FOLFOXIRI regimen (5). However, 5-FU is unstable, with a short biological half-life of 13 ± 7 min (6), and targets indifferently dividing cancer and normal cells, thus causing serious adverse effects, such as diarrhea (7.1–13.6%), nausea/vomiting (23.0%), leucopenia (2.9–12.5%), anemia (6.2%), and mucositis (14.3%) (7). Therefore, new strategies have been proposed to enhance the therapeutic index of 5-FU. Among them, 5-FU administration in nanosized carriers, such as nanoparticles, micelles, or liposomes, has been already carried out successfully (8). In particular, several studies have evaluated 5-FU entrapment in liposomal formulations with targeting ligands, such as folic acid, to overcome the drawbacks associated with passive targeting (9–13). In another approach, 5-FU co-administration with natural and safe compounds that exhibit anticancer properties, such as long-chain n-3 polyunsaturated fatty acids (n-3 PUFAs), has been proposed to reduce not only the effective therapeutic dose of 5-FU but also non-specific toxicity and cachexia associated with systemic chemotherapy (14, 15). Several studies have indeed shown that supplementation with n-3 PUFAs had a powerful adjuvant activity in combination with 5-FU (16–18). To our knowledge, however, there is no study having attempted to combine these two approaches in order to protect them from rapid degradation in the bloodstream and facilitate their simultaneous transport to the tumor site, which may be particularly relevant in the context of liver metastases (19). Therefore, the aim of this study was to encapsulate 5-FU in the aqueous core of liposomes containing n-3 PUFAs in their lipid bilayer and to evaluate *in vitro* and *in ovo* their therapeutic efficacy in different CRC models.

## MATERIALS AND METHODS

### Liposome Preparation

The classic thin film hydration method of Bangham *et al*. (20) was optimized to produce a liposomal formulation (LIPUFU) containing n-3 PUFAs in the lipid bilayer and 5-FU in the aqueous core. Docosahexaenoic acid (DHA) and eicosapentaenoic acid (EPA) (both from Chemie Brunschwig AG, Basel, Switzerland) were added at a molar ratio of 1:2 to a phospholipid mixture of 1,2-dipalmitoyl-*sn*-glycero-3-phosphocholine (DPPC), cholesterol, 1,2-dipalmitoyl-*sn*-glycero-3-phospho-(1′-*rac*-glycerol) (DPPG), and 1,2-distearoyl-*sn*-glycero-3-phosphoethanolamine-*N*-[methoxy(polyethylene glycol)-2000] (DSPE-PEG2000) (all from Corden Pharma, Liestal, Switzerland). The compounds were dissolved in 15 ml chloroform for 3 h on vortex at room temperature. A thin layer of lipid film was obtained after complete solvent evaporation under vacuum at 474 mbar for 1 h and 30 min at 43°C. The film was rehydrated in a 5-FU solution (kindly provided by the pharmacy of the Geneva University Hospital) adjusted with phosphate-buffered saline (PBS: BioConcept Ltd., Allschwil, Switzerland) at pH 7.4. Multilamellar vesicles were obtained by vortexing at room temperature for 15 min and moderate shaking on a rotavapor at 53–55°C for 30 min. The lipid suspension was then left at room temperature in the dark for 24 h. Single unilamellar vesicles of homogenous size were obtained by sonication for 5 min at a 20% amplitude (Branson Ultrasonics, Danbury, USA). After sonication, the liposomal suspension was transferred into a 15-ml Falcon tube and stored at 4°C. To remove unentrapped 5-FU, EPA, and DHA, the liposomal suspension was ultra-centrifuged at 40,000 rpm for 3 h and washed with 2 ml of water three times successively. Sonication and ultracentrifugation steps were used after early experiments showed that extrusion and dialysis steps gave low incorporation rates of 5-FU and n-3 PUFAs (data not shown). The final liposomal suspension was stored in 1× PBS at pH 7.4 after filtration through a 0.22-μm Millipore membrane.

Control liposomes without (LIP) or with only 5-FU (LIFU) or n-3 PUFAs (LIPU) were produced in a similar way.

### Liposome Characterization

Diameter, polydispersity index (PDI), and zeta potential of the different liposomal formulations were measured by photon correlation spectroscopy at 90° angle and room temperature, using a Zetasizer Nano ZS (Malvern Instruments, Ltd., UK). Liposomal suspension uniformity and morphology were further evaluated with transmission electron microscopy (Tecnai G^2^ 20 TEM, FEI Company, OR, USA). Deposit grids (formvar carbon film, 200 mesh copper grids, Electron Macroscopy Sciences, Hatfield, USA) were prepared by ionization, and 5-μl samples of the different liposomal suspensions were dropped off on them for 30 s. The grids were then washed twice in a drop of water for 2 s, dried, then left for 30 s in a drop of saturated uranyl acetate, and finally dried. Three pictures were taken at different places and magnifications for each sample.

### Loading Efficiency of 5-FU

Loading efficiency and release of 5-FU were evaluated by high-performance liquid chromatography (HPLC). 5-FU dilutions were performed to obtain a standard curve of 0.1, 0.5, 1, 1.3, 10, 100, 250, 500, and 1000 μmol/l. Liposomal suspension samples of 100 μl were lysed in 200 μl PBS with 2% Triton X-100 (Sigma-Aldrich, Buchs, Switzerland) and 5 μl 5-bromo-2′-deoxyuridine (BrUrd: Sigma-Aldrich, Buchs, Switzerland) as internal control. Ten-microliter samples were injected into a HPLC system equipped with a UV detector and data analysis software (HPLC W600 controller, W2487dual, 717 plus autosampler, Waters Corporation, MA, USA). Peak separation was performed on a C-18 reverse phase column of 3.9 × 10.5 μm (Atlantis Waters, MA, USA) at a flow rate of 1.0 ml/min and UV detection of 266 nm. The mobile phase (pH = 4.47, 25°C) consisted of 136 g NaCH_3_COO, 77 g NH_4_CH_3_COO in 1 l H_2_O, and 250 ml CH_3_COOH 10%. The retention time was 4.5 min for 5-FU and 8.8 min for BrUrd. The standard curve for 5-FU was linear from 0.013 to 130 μg/ml (*r*^2^ = 0.99). 5-FU loading efficiency was calculated according to the calibration curve, by dividing the amount measured into the liposomal suspension with the initial amount used in the liposomal formulation.

### Fatty Acid Composition of the Liposomal Membranes

Fatty acid composition of the liposomal membranes was analyzed by capillary gas chromatography (GC). Total lipids were extracted from 300-μl liposomal suspension in 6 ml chloroform-methanol 2:1 (v:v) containing 0.1% butylated hydroxytoluene. Diphosphatidyl margaric acid was added as internal standard. The extract was washed with 1.5 ml NaCl and the mixture was vortexed 10 min at 2000 rpm and centrifuged 10 min at 4000 rpm. The organic phase was transferred into glass tubes with screw caps and evaporated for 35 min at 45°C and 275 mbar, using the RapidVap® system (VWR International LLC, PA, USA). The lipid extract was resuspended in 80 μl dichloromethane, and 25 μl Methyl-Prep II (Grace Davison, Bannockburn, IL) was added to the mixture before incubation for 10 min at room temperature to allow fatty acid conversion into the corresponding fatty acid methyl esters (FAMEs). FAMEs were then extracted by evaporation for 5 min at 40°C and 250 mbar, using the RapidVap® system, and resuspended in 300 μl iso-octane/butylated hydroxytoluene. After vortexing for 10 s, the suspension was centrifuged for 2 min at 5000 rpm and the supernatant was transferred into autosampler vials. FAME composition was determined by injecting 1-μl samples through the split injector (ratio 25) at 60°C into a GC device equipped with an automatic injector (gas chromatograph: 430-GC, Bruker Daltonik GmbH, Bremen, Germany) operating at detector temperatures of 250°C. A Select FAME column of 50 m × 0.25 mm ID × 0.25-mm film thickness was used for FAME separation (Agilent Technologies, Santa Clara, USA). Hydrogen was used as the carrier gas at a flow rate of 2.8 ml/min, with nitrogen as the make-up gas for the flame ionization detector. The temperature ramp was programmed to rise from 60 to 250°C at a rate of 12°C/min and then kept constant for 7 min at 250°C to wash the column. FAME identification was obtained by comparison with the retention times of pure standard mixtures. The retention time was 15.38 and 16.71 min for EPA and DHA, respectively. The fatty acid amount (μmol/l) was quantified by integrating the peak and adjusting the results with the internal standard. Incorporation efficiency of DHA and/or EPA was calculated by dividing the amount actually incorporated into the liposomal suspension with the initial amount used in the liposomal formulation.

### Cell Lines and Culture

Two human colorectal adenocarcinoma cell lines, LS174T (ATCC no. CL-188™, USA) and HT-29 (ATCC no. HTB-38™, USA), were chosen according to their different genetic background and sensitivity to 5-FU. According to Violette *et al*. (21), LS174T (p53^+/+^, bax^−/−^) shows greater resistance to 5-FU than HT-29 (p53^−/0^, bax^+/+^). They were kept in exponential growth phase at 37°C and 5% CO_2_ by subculturing twice a week in Dulbecco’s modified Eagle’s medium supplemented with 10% heat-inactivated fetal bovine serum and 0.1 g/l penicillin-streptomycin (all from Invitrogen, Zug, Switzerland).

### Cytotoxicity Assays

The cytotoxic potential of the different liposomal formulations was evaluated on the two cell lines, using fluorescence-activated cell sorting (FACS) analyses. Cells were seeded 24 h before treatment at a density of 20,000 cells/well in 24-well plates (BD Biosciences, Allschwil, Switzerland). After treatment, cells were detached with 0.2 ml trypsin-EDTA 1× (Life Technologies, Zug, Switzerland), washed with 1 ml PBS 1×, and centrifuged for 10 min at 1200 rpm. The pellet was then resuspended in 2 μg/0.4 ml propidium iodide (PI: BD Biosciences, Allschwil, Switzerland) to allow discrimination between permeable cells labeled with PI (dead) and unlabeled cells (living). The number of PI-labeled and unlabeled cells per microliter was then counted using a BD Accuri C6 flow cytometer (BD Biosciences, Allschwil, Switzerland) at excitation and emission wavelengths of 488 and 530 nm, respectively. FACS analysis was carried out with the corresponding Accuri C6 software.

### Cell Cycle Modulation

One of the main mechanisms of action of 5-FU is to block the cell cycle in S phase by inhibition of the thymidylate synthase. Therefore, the cytostatic effect of the liposomal emulsions was also evaluated by adding to the previous protocol a denaturation step before PI staining of the nuclei. After detachment and centrifugation, cells were fixed by adding, drop by drop while vortexing, 0.4 ml of cold 70% ethanol into the cell pellet and then stored > 2 h at − 20°C. At the day of analysis, cells were washed twice to remove the ethanol and resuspended in PI/RNase staining buffer (BD Biosciences, Allschwil, Switzerland) at a concentration of 10^6^ cells/0.5 ml. Cells were then incubated 15 min in the dark at room temperature and stored at 4°C before FACS analysis as previously described.

### Apoptosis Induction

Apoptosis induction by the different liposomal suspensions was quantified using a two-parameter FACS analysis with annexin V/PI detection kit according to the manufacturer’s instruction (BD Biosciences, Allschwil, Switzerland). Briefly, cells were detached with trypsin-EDTA 1×, washed with PBS 1×, and then resuspended in binding buffer (10 mM HEPES/NaOH (pH 7.4), 140 mM NaCl, 2.5 mM CaCl_2_) at a concentration of 1 × 10^6^ cells/ml. Samples were stained with 5 μl annexin V conjugated with fluorescein isothiocyanate (FITC) and 5 μl PI at room temperature for 15 min in the dark. They were then diluted in 0.4-ml binding buffer and analyzed within 1 h using a BD Accuri C6 flow cytometer at excitation and emission wavelengths of 488 and 530 nm, respectively.

### Chorioallantoic Membrane Assay

A first *in vivo* evaluation of the antitumor potential of the liposomes was performed on human CRC tumors xenografted on chorioallantoic membranes (CAMs) of chick embryos lacking B and T cell–mediated immunity (22). Fertilized chick eggs were placed on rotating grids in an incubator (37°C, 60% humidity), with the narrow apex down for 3 days. At embryo development day 3 (EDD3), a hole was drilled into the eggshell at the narrow apex, covered with adhesive tape to avoid dehydration, and returned into the incubator. At EDD7, the hole was enlarged to 1–2 cm. With a needle, CAM was scratched close to a blood vessel or around a junction of several blood vessels, and a silicon O-ring (Apple Rubber Products Inc. Lancaster, USA) was deposited at this place. Before implantation in the O-ring, cells were treated with 0.5% trypsin-EDTA 1×. The last resuspension was done in a nutriment solution containing 50% Matrigel (BD Biosciences, Belford, USA)/50% medium in order to obtain a concentration of 5 × 10^6^ cells in 20 μl. To avoid desiccation and contamination, the window on the eggshell was sealed with parafilm and the eggs were returned into the incubator until the day of treatment. At EDD 12, either a sham treatment with PBS or the LIPUFU suspension was injected i.v. into the main blood vessel through a 33″-gauge needle at a volume of 25 μl. Tumor growth was assessed at 24 h, 48 h, and 72 h after PBS or LIPUFU injection by means of image recording and bi-dimensional measurements of the tumor size using a binocular microscope (Leica M205FA microscope, objective × 10, FOV: 1.5052 mm^2^).

### Statistical Analysis

Every experiment was performed in quadruplicate samples. The variables were expressed as proportions or means ± 1 standard deviation (SD), as appropriate. Differences between the different treatment conditions were analyzed with one-way ANOVA followed by a post hoc Tukey’s multiple comparisons test after checking the normal distribution and equality of variance of the data with the skewness and kurtosis test and the Bartlett’s test, respectively. In case of unequal variance, the Kruskal-Wallis test was used, followed by the two-sample *t* test for comparison between two treatment conditions. All statistical analyses were performed with the Stata/IC 13.1 software for Windows (StataCorp LP, College Station, TX, USA). Statistical significance is reported as follows: *, *P* < 0.05; **, *P* < 0.01; ***, *P* < 0.001.

## RESULTS

### Liposomal Formulation

**Fig. 1:**
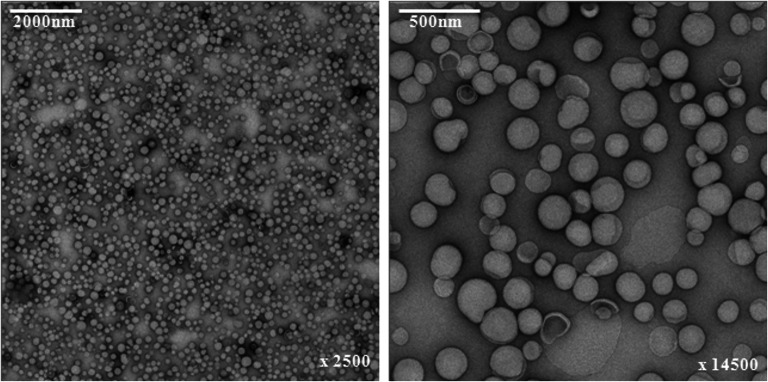
Transmission electron micrographs of LIPUFU formulation composed of DPPC, DPPG, cholesterol, DPPG, DPA, and EPA with 5-FU encapsulation at 2000 and 500 nm

Different preparation procedures were evaluated to obtain stable liposomes of uniform size with an optimal concentration of n-3 PUFAs and 5-FU. The most favorable LIPUFU formulation was obtained by adding 2.5 mg DHA and 5 mg EPA at a ratio of 1:2 to a phospholipid mixture of 78 mg DPPC, 64 mg cholesterol, 4.2 mg DPPG, and 2.1 mg DSPE-PEG2000. The rehydration solution consisted of 0.65 mg 5-FU in 5 ml PBS at pH = 4. This formulation gave liposomes with uniform size of 154 nm, PDI of 0.19, and zeta potential of − 42 mV (Fig. 1 and Table I). As evaluated by GC analysis, the incorporation rate of DHA and EPA into the lipid bilayer was 37% and 44%, respectively, corresponding to 557 μmol/l DHA and 1467 μmol/l EPA. HPLC analysis showed that the entrapment rate of 5-FU in the aqueous core of the liposomes was only 1% corresponding to 9.8 μmol/l 5-FU (Table I).

**Table I:** Description of the Different Liposomal Formulations

Type	Nb	Size (nm)	PDI	Zeta (mV)	5-FU (μmol/l)			DHA (μmol/l)			EPA (μmol/l)			Ratio
		Mean ± SD	Mean ± SD	Mean ± SD	Initial	Final	(%)	Initial	Final	(%)	Initial	Final	(%)	DHA/EPA
LIP	3	146 ± 5	0.15 ± 0.02	− 41 ± 4		-			-			-		-
LIFU	3	155 ± 9	0.18 ± 0.05	− 40 ± 5	1000	7.1 ± 1.8	(0.7)		-			-		-
LIPU	3	150 ± 3	0.16 ± 0.02	− 43 ± 3		-		1522	601 ± 156	(39.5)	3306	953 ± 458	(28.8)	0.63
LIPUFU	4	154 ± 4	0.19 ± 0.03	− 41 ± 2	1000	9.8 ± 1.1	(1.0)	1522	557 ± 210	(36.6)	3306	1467 ± 362	(44.4)	0.38

*SD* standard deviation, *PDI* polydispersity index, *mV* millivolts, *5-FU* 5-fluorouracil, *DHA* docosahexaenoic acid, *EPA* eicosapentaenoic acid, *LIP* liposome without 5-fluorouracil and n-3 polyunsaturated fatty acids, *LIFU* liposome with 5-fluorouracil, *LIPU* liposome with n-3 polyunsaturated fatty acids, *LIPUFU* liposomes with 5-fluorouracil and n-3 polyunsaturated fatty acids, Nb number

### LIPUFU Cytotoxicity

**Fig. 2:**
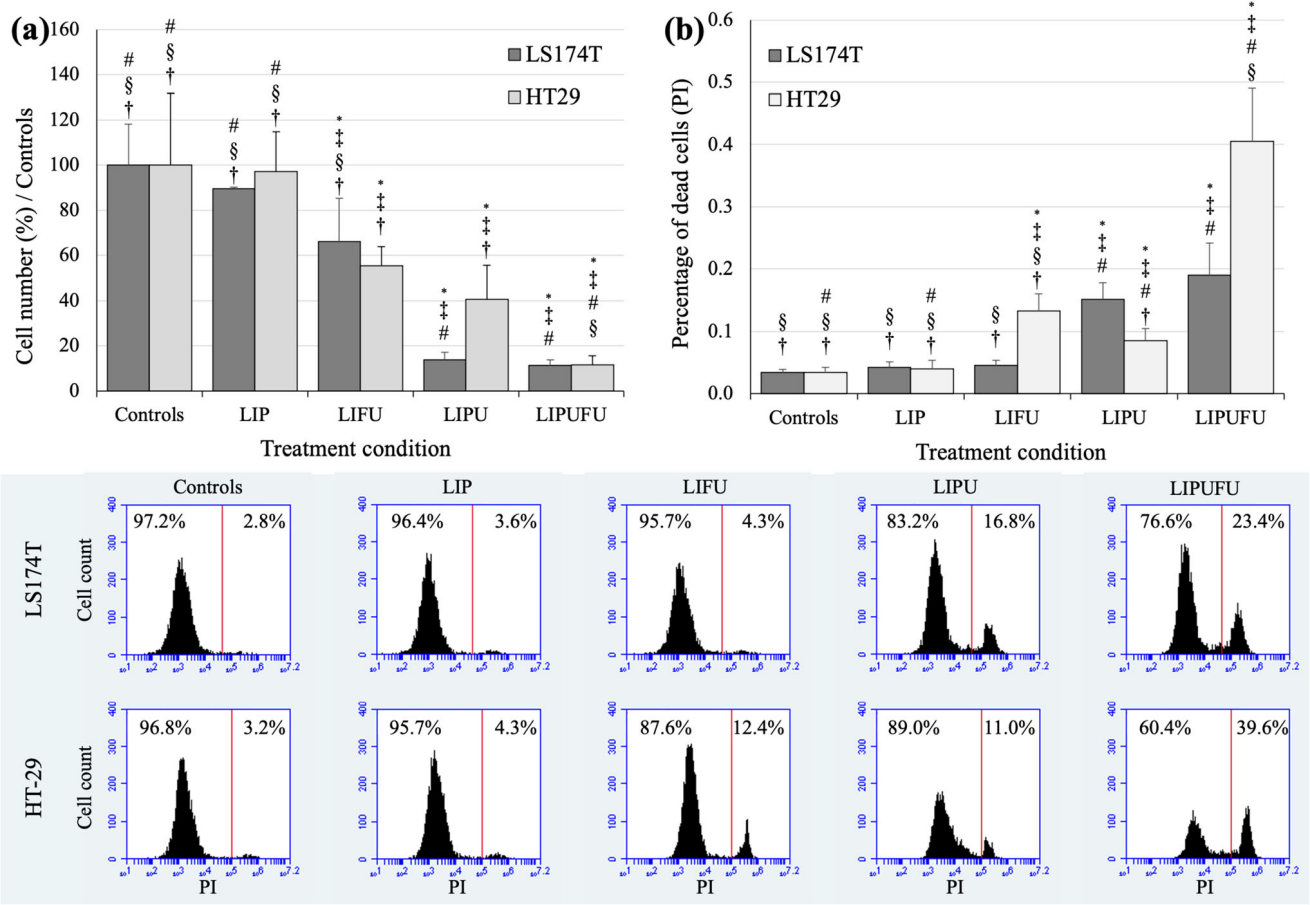
Effect of a 48-h incubation in 1/20 dilution of LIP, LIFU, LIPU, or LIPUFU on the cell growth (**a**) and viability (**b**) of LS174T and HT-29 cells, as measured by fluorescence-activated cell sorting analysis with PI staining of the nuclei of dead cells. Columns represent means ± 1 SD of 4 samples/condition. *, different from controls; ^‡^, different from LIP; ^#^, different from LIFU; ^§^, different from LIPU; ^†^, different from LIPUFU (*t* test, *P* ≤ 0.05)

The cytotoxic potential of LIPUFU was compared with that of LIP, LIFU, and LIPU in the two HT-29 and LS174T cell lines. While LIP had no effect compared to untreated controls, LIPUFU, and to a lesser extent LIFU and LIPU, significantly decreased the growth (Fig. 2a) and viability (Fig. 2b) of both cell lines. As expected, HT-29 cells were more sensitive to LIFU containing 5-FU than LS174T cells, while LS174T cells appeared to be more sensitive to LIPU containing n-3 PUFAs than HT-29 cells. LIPUFU was more cytotoxic than LIP and LIFU in both cell lines (*P* < 0.01) and LIPU in HT-29 cells (*P* < 0.001) (Fig. 2).

### Cell Cycle Modulation

**Fig. 3:**
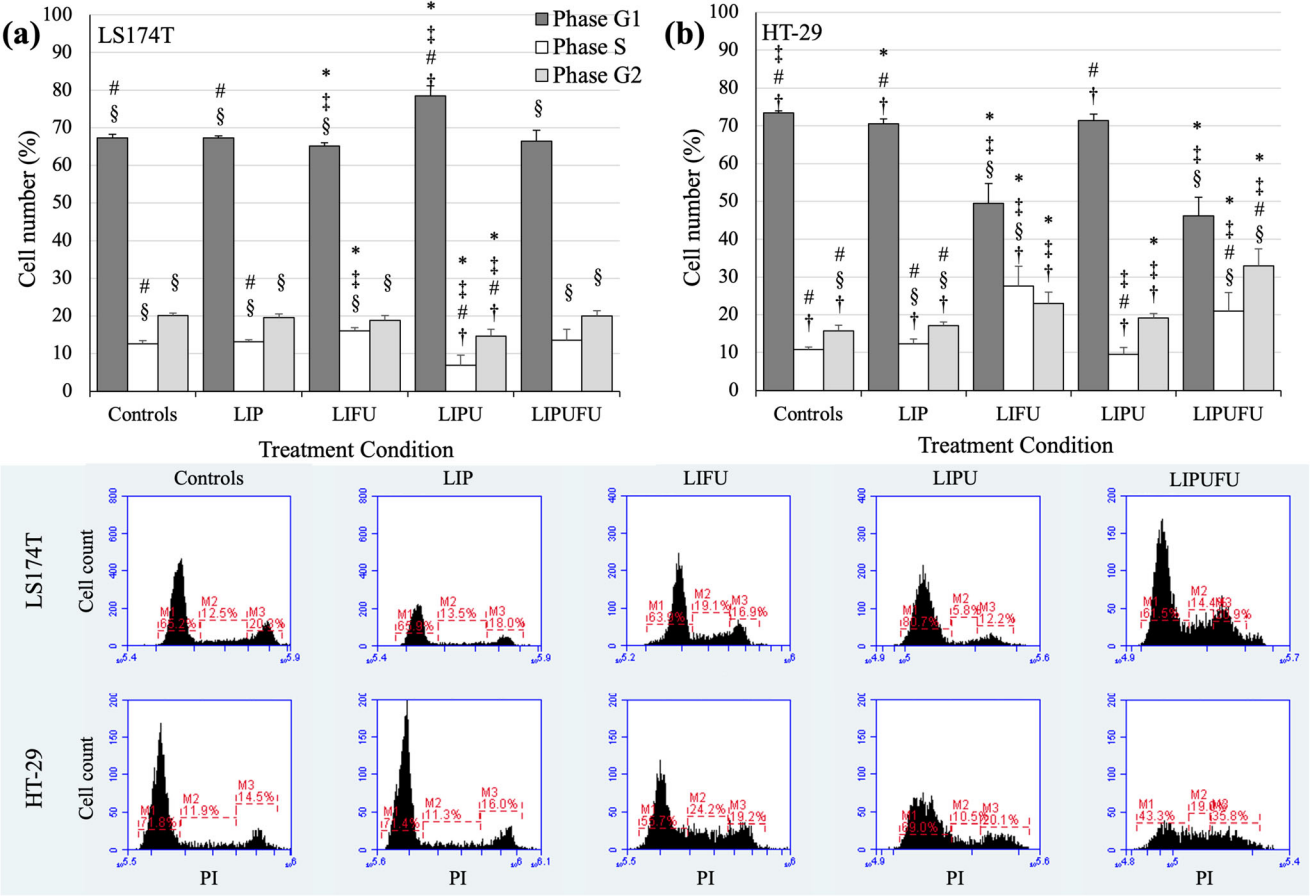
Effect of a 24-h incubation in 1/20 dilution of LIP, LIFU, LIPU, or LIPUFU on the cell cycle of LS174T (**a**) and HT-29 (**b**) cells, as measured by fluorescence-activated cell sorting analysis with PI staining of the DNA content of cell nuclei. Columns represent means ± 1 SD of 4 samples/condition. *, different from controls; ^‡^, different from LIP; ^#^, different from LIFU; ^§^, different from LIPU; †, different from LIPUFU (*t* test, *P* ≤ 0.05)

Since 5-FU blocks the cell cycle in the S phase, the cytostatic potential of LIP, LIFU, LIPU, and LIPUFU was evaluated in LS174T (Fig. 3a) and HT-29 (Fig. 3b) cells. As expected, LIFU and LIPUFU acted similarly in both cell lines by significantly decreasing the percentage of cells in the G1 phase and increasing the percentage of cells in the S phase, whereas LIPU containing the n-3 PUFAs seemed to act differently by blocking LS174T cells in the G1 phase, but had no effect on HT-29 cell cycle.

### Apoptosis Induction

An increase in the percentage of apoptotic cells was only observed in HT-29 cells treated with LIFU (4.8 ± 0.7%, *P* = 0.002) or LIPUFU (3.3 ± 0.5%, *P* = 0.001) compared to untreated controls (1.8 ± 0.2%). LIPUFU increased the percentage of necrotic cells in both LS174T (10.3 ± 3.3% *vs* 5.1 ± 1.0%, *P* = 0.02) and HT-29 (14.5 ± 0.7% *vs* 3.1 ± 2.7%, *P* = 0.004) cells compared to untreated controls.

### *In Vivo* Tumor Toxicity

**Fig. 4:**
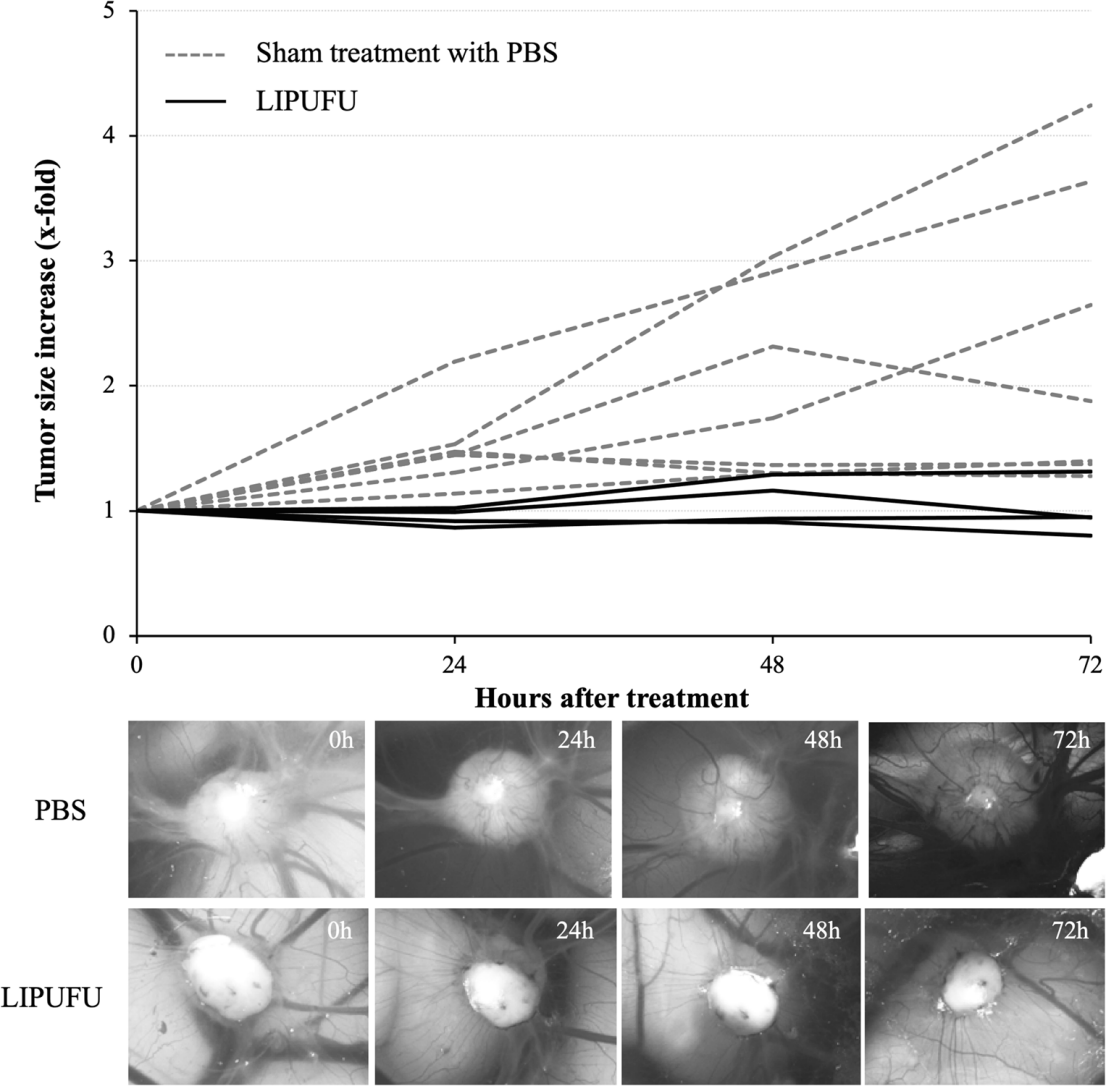
Growth of HT-29 xenografted tumors on the CAM after an i.v. injection of 25 μl of LIPUFU (*n* = 4) or a sham treatment of PBS (*n* = 7), as measured from the two main axes of the tumors (two-way ANOVA: *F*(4,92), *P* = 0.0006). Tumor growth at 72 h was significantly different between the two treatments (post hoc Tukey’s multiple comparisons test: contrast = − 1.347-fold, *P* = 0.02)

A first *in vivo* assessment of the antitumor potential of LIPUFU was performed in the CAM model. Since it was not possible to obtain solid LS174T tumors in this model, only the growth of HT-29 tumors was measured over a 3-day period after a sham treatment with PBS or with LIPUFU. Overall, PBS-treated tumors grew more or less rapidly, whereas LIPUFU-treated tumors tended to not grow or even decrease in size (Fig. 4).

## DISCUSSION

**Fig. 5:**
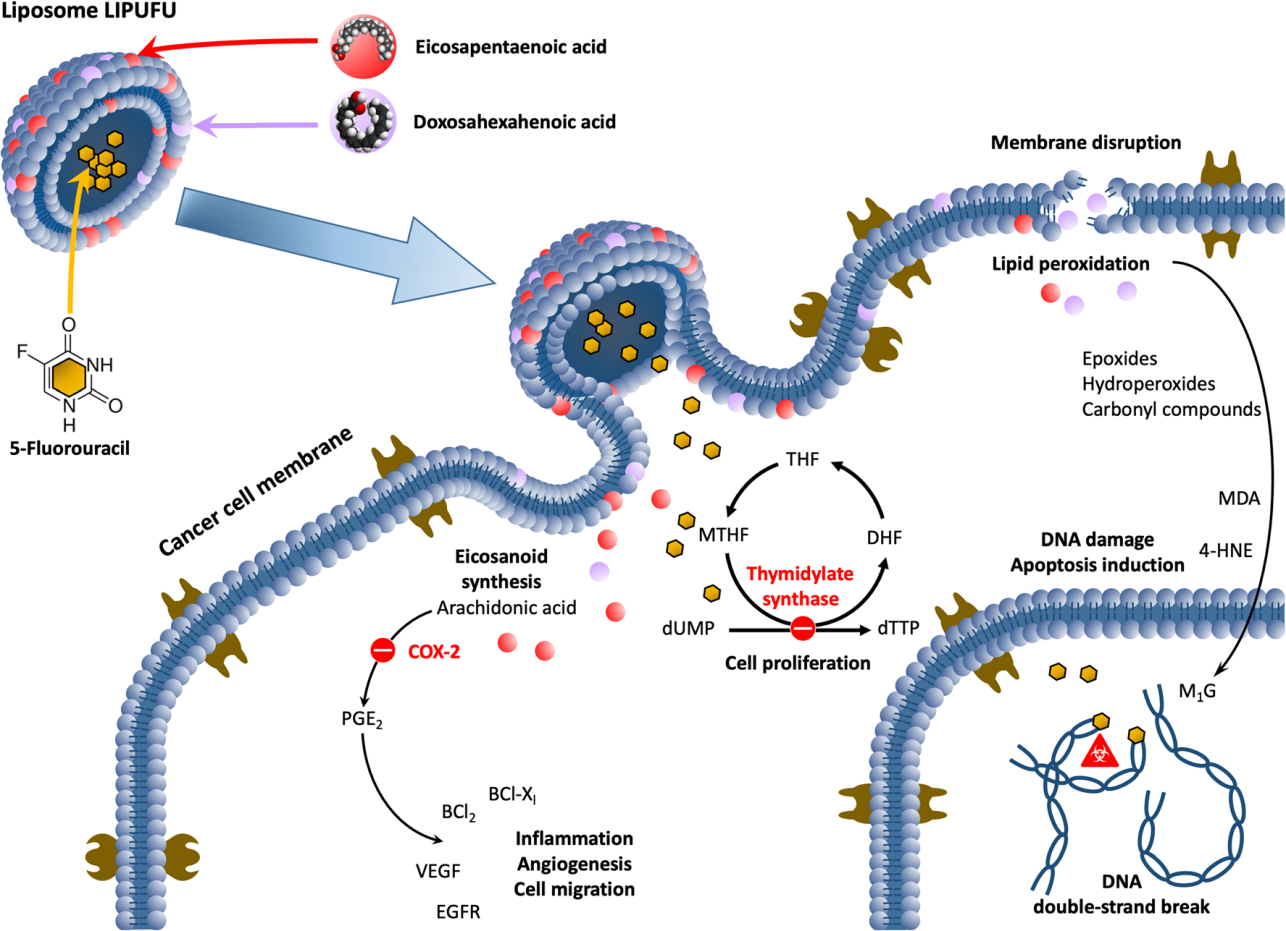
Schematic diagram of the possible mechanisms of action allowing n-3 polyunsaturated fatty acids to act in synergy with 5-fluorouracil on cancer cells. While 5-fluorouracil induces cell cycle arrest and pyrimidine misincorporation into DNA, eicosapentaenoic acid and docosahexaenoic acid hamper inflammation, angiogenesis, and cell migration through competitive inhibition of cyclooxygenase II metabolic pathway and induce DNA damage and cell membrane disruption through their peroxidation products. COX-2, cyclooxygenase II; DHF, dihydrofolate; dTTP, thymidine triphosphate; dUMP, deoxyuridine monophosphate; EGFR, epidermal growth factor receptor; 4-HNE, 4-hydroxynonenal; MDA, malondialdehyde; M1G, pyrimido[1,2-a]purin-10(3H)-one; MTHF, methyltetrahydrofolate; PGE2, prostaglandin E2; THF, tetrahydrofolate; VEGF, vascular endothelial growth factor

Any enhancement of the therapeutic index of 5-FU would be of great value in the management of advanced CRC patients. Among the various strategies envisaged, 5-FU entrapment in synthetic carriers has been evaluated with more or less success to facilitate its delivery to tumor site (10, 23–25). Fanciullino *et al*. could enhance the antitumor effect of 5-FU and the lifespan of animals xenografted with CRC tumors by entrapping 5-FU with a putative modulator, 2′-deoxyinosine, in a double-liposomal formulation (26). However, 5-FU is a very membrane-permeable drug that is poorly retained within the aqueous liposomal core. This limitation was of great concern in the present study because the presence of long-chain n-3 PUFAs into the lipid bilayer had the effect of fluidizing the liposomal membranes. Therefore, we optimized the formulation to facilitate the entrapment of 5-FU and n-3 PUFAs in liposomes under 150 nm of size. In the literature, it is reported that pH change may help 5-FU entrapment when basic solution is used for rehydration rising from 7% at neutral pH to 10% in basic pH (27). On the other hand, Fanciullino *et al*. obtained entrapment rates of 10 to 25%, without however observing any difference whether the pH was neutral or basic (26, 28). Therefore, we maintained a neutral pH for the rehydration of the phospholipid bilayer. Incorporation of n-3 PUFAs in the lipid bilayer was low compared to the literature (from 35 to 73.5% (29, 30)). This is likely related to the addition of cholesterol in our formulation. Indeed, most of the studies about n-3 PUFA incorporation in liposomes did not use cholesterol in the formulation because of the antagonism effect of these two compounds. Whereas cholesterol tends to rigidify the membrane, n-3 PUFAs are known to fluidize the lipid bilayer. As regards our study, n-3 PUFAs could have facilitated 5-FU release out of the liposome. Therefore, a balance had to be found between n-3 PUFAs and cholesterol incorporation to allow 5-FU entrapment in the aqueous core. Even so, the entrapment rate of 5-FU has remained low and could be improved in the future by other strategies, such as ternary metal complexation (31). Our liposomal formulation contained DHA and EPA at a molar ratio of 1:2 to promote a possible immunomodulatory effect on cyclooxygenase II activity (32). However, DHA and EPA did not incorporate at the same ratio whether or not 5-FU was present, possibly because these molecules do not have the same flexibility and were reported to have distinct membrane locations and lipid interactions (33). Nevertheless, the present liposomal formulation was shown to be more cytotoxic than 5-FU or n-3 PUFAs entrapped alone in similar liposomes. Moreover, LIPUFU proved to be as effective on a resistant cell line (LS174T) as on a 5-FU-sensitive one (HT29). The cytostatic effect of 5-FU seemed to be enhanced by n-3 PUFAs, since LIPUFU blocked the cell cycle in S phase in a similar way as LIFU, whereas LIP and LIPU did not reproduce the same effect. A plethora of mechanisms have been put forward to explain the synergistic effect of n-3 PUFAs on CRC chemotherapy (15). The main ones are shown in Fig. 5. Among them, the presence of n-3 PUFAs in the membranes may alter lipid raft behavior and increase lipid peroxidation (14). Excessive oxidative stress in CRC cells may induce apoptosis and necrosis by damaging cellular components such as DNA, protein, and membranes (34, 35). In particular, the peroxidation products of n-3 PUFAs may contribute to genetic instability together with 5-FU by causing nucleotide oxidation and generation of alkali-labile sites (36). This hypothesis is supported by a study demonstrating that liposomes loaded with 5-FU and the antioxidant apigenin could induce cell cycle arrest and apoptosis of HT-29 and HTC-15 cells (37). Another mechanism may involve downregulation of COX-2-dependent synthesis of PGE2 by n-3 PUFAs, thus inducing apoptosis through a Bax-dependent mitochondrial pathway (16). This modulation pathway is undoubtedly particularly relevant in the context of CRC cells where COX-2 is overexpressed (38). The antitumor effect of n-3 PUFAs could be further amplified *in vivo* by downregulation of VEGF and EGFR synthesis and thus inhibition of angiogenesis and cell migration (39). We therefore performed a preliminary evaluation of the antitumor effect of LIPUFU on CRC tumors xenografted in the CAM model and observed that a single injection of LIPUFU could effectively inhibit tumor growth. Although the use of this model was intended to avoid animal sacrifice, it was found to be limited by the difficulty in obtaining tumors of uniform size, in particular with the LS174T cell line, and by the impossibility of evaluating tumor growth inside the CAM. Thus, this first results need confirmation in another animal model allowing tri-dimensional evaluation of the tumor growth and possible side effects on body weight, blood cell number, and inflammatory parameters.

## CONCLUSION

5-FU-loaded liposomes containing n-3 PUFAs were successfully synthesized with a good reproducibility. Although 5-FU and n-3 PUFA entrapments were not optimal, a strong cytotoxic effect was observed on both HT29 and LS174T cell lines. These first results indicate that such an approach could be envisaged in CRC chemotherapy to reduce the effective therapeutic dose and thus toxicity of 5-FU on healthy tissues with rapid cellular turnover.

## Abbreviations

BrUrd: 5-bromo-2′-deoxyuridine
CAM: chicken chorioallantoic membranes
CRC: colorectal cancer
DHA: docosahexaenoic acid
DPPC: 1,2-dipalmitoyl-*sn*-glycero-3-phosphocholine
DPPG: 1,2-dipalmitoyl-*sn*-glycero-3-phospho-(1′-rac-glycerol)
DSPE-PEG2000: 1,2-distearoyl-*sn*-glycero-3-phosphoethanolamine-*N*-[methoxy(polyethylene glycol)-2000]
EDD: embryo development day
EPA: eicosapentaenoic acid
FACS: fluorescence-activated cell sorting
FAME: fatty acid methyl esters
FITC: fluorescein isothiocyanate
5-FU: 5-fluorouracil
GC: gas chromatography
HPLC: high-performance liquid chromatography
LIFU: liposome with 5-FU
LIP: liposome without 5-FU and n-3 PUFAs
LIPU: liposome with n-3 PUFAs
LIPUFU: liposomes with 5-FU and n-3 PUFAs
PBS: phosphate-buffered saline
PDI: polydispersity index
PI: propidium iodide
n-3 PUFAs: n-3 polyunsaturated fatty acids
TEM: transmission electron microscopy
